# The ever unfolding story of cAMP signaling in trypanosomatids: *vive la difference*!

**DOI:** 10.3389/fphar.2015.00185

**Published:** 2015-09-07

**Authors:** Daniel N. A. Tagoe, Titilola D. Kalejaiye, Harry P. de Koning

**Affiliations:** ^1^Wellcome Trust Centre for Molecular Parasitology, University of Glasgow, Glasgow, UK; ^2^Institute of Infection, Inflammation and Immunity, College of Medical, Veterinary and Life Sciences, University of Glasgow, Glasgow, UK; ^3^Department of Laboratory Technology, Division of Medical Laboratory Technology, University of Cape Coast, Cape Coast, Ghana

**Keywords:** *Trypanosoma cruzi*, *Trypanosoma brucei*, *Leishmania*, phosphodiesterase, cAMP, kinase, adenylyl cyclase, PKA

## Abstract

Kinetoplastids are unicellular, eukaryotic, flagellated protozoans containing the eponymous kinetoplast. Within this order, the family of trypanosomatids are responsible for some of the most serious human diseases, including Chagas disease (*Trypanosoma cruzi*), sleeping sickness (*Trypanosoma brucei* spp.), and leishmaniasis (*Leishmania* spp). Although cAMP is produced during the life cycle stages of these parasites, its signaling pathways are very different from those of mammals. The absence of G-protein-coupled receptors, the presence of structurally different adenylyl cyclases, the paucity of known cAMP effector proteins and the stringent need for regulation of cAMP in the small kinetoplastid cells all suggest a significantly different biochemical pathway and likely cell biology. However, each of the main kinetoplastid parasites express four class 1-type cyclic nucleotide-specific phosphodiesterases (PDEA-D), which have highly similar catalytic domains to that of human PDEs. To date, only TbrPDEB, expressed as two slightly different isoforms TbrPDEB1 and B2, has been found to be essential when ablated. Although the genomes contain reasonably well conserved genes for catalytic and regulatory domains of protein kinase A, these have been shown to have varied structural and functional roles in the different species. Recent discovery of a role of cAMP/AMP metabolism in a quorum-sensing signaling pathway in *T. brucei*, and the identification of downstream cAMP Response Proteins (CARPs) whose expression levels correlate with sensitivity to PDE inhibitors, suggests a complex signaling cascade. The interplay between the roles of these novel CARPs and the quorum-sensing signaling pathway on cell division and differentiation makes for intriguing cell biology and a new paradigm in cAMP signal transduction, as well as potential targets for trypanosomatid-specific cAMP pathway-based therapeutics.

## Introduction

Trypanosomatids are protozoan parasites belonging to the order kinetoplastida, family trypanosomatidae, and are characterized by a particular substructure of the mitochondrion, called the kinetoplast, which contains the mitochondrial DNA. They are digenetic flagellated protozoans with similar cellular structures as well as similar genome organization and are all known to undergo morphological transformations during their life cycles (Stuart et al., 2008). Members of this order are unicellular eukaryotes, and many of them parasitize multicellular organisms and cause medically and economically important diseases in humans, their domestic animals and cash crops (Barrett et al., 2003). Human African trypanosomiasis (HAT), also known as African sleeping sickness, is a vector-borne parasitic disease caused by the protozoan pathogen *Trypanosoma brucei* and transmitted by several *Glossina* species, commonly called tsetse flies (Stich et al., 2002). There are three sub-species of *T. brucei* that infect mammals: *Trypanosoma brucei brucei*, *Trypanosoma brucei gambiense*, and *Trypanosoma brucei rhodesiense*. However, only *T. b. gambiense* (acute infections) and *T. b. rhodesiense* (chronic infections) infect and cause clinical disease in humans whilst *T. b. brucei* infect animals causing the disease known as nagana in cattle (Fevre et al., 2006; Brun et al., 2010). It has been estimated that the core group of neglected tropical diseases (NTDs), the majority of which are caused by trypanosomatids, results in the loss of more than 57 million disability-adjusted life years (DALY), coupled with attendant impacts on poverty (Hotez et al., 2006, 2009). Although there has been dramatic improvement in infections and death in recent years (Simarro et al., 2011), optimism is tempered in the light of previous recurrences, migration and the instability in many endemic regions (Odiit et al., 2005; Picozzi et al., 2005; Mumba et al., 2011; Blum et al., 2012). Although the old, toxic and difficult to administer drugs have helped to combat the disease until the present (Delespaux and de Koning, 2007; Kennedy, 2008; Barrett, 2010; Jacobs et al., 2011), the current increase in resistance to these drugs is very worrying (Vincent et al., 2010; Simarro et al., 2012; Baker et al., 2013). If modern standards in pharmacology were to be applied, the aforementioned issues with trypanosomatid chemotherapy mean that there are effectively no acceptable chemotherapies for these diseases. A quest to produce more clinically effective and less toxic drugs is hampered by the fact that, as eukaryotes, trypanosomatids are genetically and evolutionarily much closer to their human hosts than bacteria, resulting in problems with selectivity and toxicity (Seebeck et al., 2011). In the context of cAMP metabolism, the kinetoplastid phosphodiesterases (PDEs) are highly similar to that of most of the well-studied human homologs. However, PDEs are highly amenable to selective inhibition, due to small differences in their binding pockets that can be exploited by structure-based inhibitor design, even when using the pharmacologically well explored scaffolds of human PDE inhibitors. Moreover, downstream effectors of cAMP are very different in human and trypanosomatid cells, potentially providing further drug targets, this time without mammalian counterparts.

## Signal Transduction in Trypanosomatids

### Adenylate Cyclases

Signal cascades exist for the amplification of a small signal into a large response, leading to significant cellular changes such as expression of specific genes, the activity of certain proteins, or changes in cell cycle progression. Many disease processes, such as diabetes, heart disease, autoimmunity and cancer, arise from defects in signal transduction pathways, further highlighting the critical importance of signal transduction to biology as well as the development of medicine (Huang et al., 2010). Cyclic AMP levels in most eukaryotes are increased by stimulation of adenylyl cyclases (ACs), whilst cyclic nucleotide PDEs degrade the phosphodiester bond in cAMP, thereby limiting or abrogating signal transduction. A putative kinetoplastid AC gene was first identified in *T. brucei* when the active gene expression site of a variant surface glycoprotein (VSG) was sequenced, revealing that there were multiple genes in the site that were co-expressed with VSG. These genes were termed expression site-associated genes (ESAGs), and one of them, ESAG4, showed homology with an AC from yeast (Pays et al., 1989). Further copies of apparent ACs were identified in the genome and named GRESAG4.1 and GRESAG4.2 (genes related to ESAG4; Pays et al., 1989). Related genes were also found in *T. b. gambiense*, *Trypanosoma congolense*, *Trypanosoma mega*, *Trypanosoma equiperdum*, and *Trypanosoma vivax*. These apparent AC genes were proven to actually code for functional AC enzymes by complementing AC-deficient yeast mutants (Ross et al., 1991; Paindavoine et al., 1992).

Since then similar multigene families with high homology to ESAG4 and GRESAG4.1 have also been identified in *Leishmania donovani* and *T. cruzi*, and these ACs share the same predicted protein architecture (Sanchez et al., 1995). *T. brucei* encodes up to 20 telomeric ESAG4 AC genes and approximately 65 GRESAG4 proteins (Salmon et al., 2012a,b) and at least some of these are localized along the flagellum both in the mammalian-infective bloodstream forms (BSFs) and in procyclic (fly midgut stage) cells (Paindavoine et al., 1992; Saada et al., 2014). Their similarity results in cross-reactivity with some antibodies raised against ESAG4 (Paindavoine et al., 1992; Oberholzer et al., 2011). Whereas knocking out ESAG4 from the expression site does not affect parasite proliferation, a knockdown of all the AC family that includes ESAG4 and the two GRESAG4 genes led to a total decrease in AC activity, resulting in a phenotype that is defective in cytokinesis (Salmon et al., 2012a).

Trypanosomatid ACs contain a single *trans*-membrane domain, a conserved intracellular C-terminal domain, and a large variable extracellular domain. The N-terminal domains may function as receptors, similar to mammalian receptor-type guanylyl cyclases (Garbers et al., 2006). Whilst the catalytic domain is structurally very similar to those of mammalian ACs, it is not activated by forskolin. The purified proteins form homodimers *in vitro* (Bieger and Essen, 2001; Naula et al., 2001; Gould and de Koning, 2011) and dimerization was recently also shown *in vivo* (Saada et al., 2014). The possibility of the N-terminal extracellular domain of ACs acting as a receptor for signaling due to the lack of G-protein-coupled receptor (GPCR) in the kinetoplastid genome has been strongly speculated (Seebeck et al., 2004; Laxman and Beavo, 2007). Indeed, the recently revealed relationship of the N-terminal of a representative AC from *T. brucei* with an *Escherichia coli* L-leucine-binding protein (LBP; Emes and Yang, 2008) and a similar LBP that acts as an amide receptor in *Pseudomonas aeruginosa* (O’Hara et al., 2000) have lend support to the hypothesis that AC activity could be directly regulated by extracellular stimuli. Analysis of the AC gene clusters also showed that the variation in the extracellular domains, specifically in areas predicted to come into close contact with putative ligands, appear to be significantly driven by positive selection. This is an indicator of adaptive evolution and consistent with a receptor or sensory function for at least some of the cyclases (Emes and Yang, 2008). Although no putative ligand has been identified as yet, some extracts from *T. cruzi* and *T. brucei* insect vectors have been shown to activate ACs, whilst a low-molecular-weight molecule, stumpy induction factor (SIF), probably secreted by the trypanosome itself, was inferred to trigger the differentiation of long slender bloodstream *T. brucei* to the non-replicating stumpy form via the cAMP signaling cascade (Garcia et al., 1995; van den Abbeele et al., 1995; Vassella et al., 1997). In addition it has been shown that ACs influence host parasite interactions through the modulation of tumor necrosis factor alpha (TNF-*α*) and that AC activity of lysed trypanosomes contributes to establishing an infection of these parasites in a host (Salmon et al., 2012b).

The above suggests that diversity in ACs provides an adaptive advantage to the extracellular *T. brucei* enabling host immune evasion and modulation, and thus survival. It is postulated that the high number of ACs of *T. brucei* species, compared with the intracellular *T. cruzi* and *Leishmania*, has to do with the evasion of the host immune system by continuously switching its VSG expression in the various telomeric sites, which resulted in the duplication of ESAGs, including ACs (ESAG4). Other forces of selection may then have resulted in alternative specificities for the individual receptor-cyclases to arise. Thus, the large numbers of ACs found in the *T. brucei* genome may allow more specific responses to the multiple ligands found in its extracellular environment, compared with the relatively sheltered intracellular lifestyle of *Leishmania* and *T. cruzi* (Gould and de Koning, 2011).

The cellular localization of the ACs of kinetoplastids is also consistent with them acting as a receptor. Antibodies against ESAG4 were shown to specifically bind to the cell surface along the flagellum in both BSFs and procyclic trypanosomes (Paindavoine et al., 1992). Similarly, in *T. cruzi* epimastigotes, the calcium-stimulatable AC was found to be associated with the flagellum (D’Angelo et al., 2002).

Proteomic analysis of bloodstream *T. brucei* flagella and plasma membrane fractions have identified receptor and transport-like proteins that likely play important roles in signaling and parasite-host interactions (Bridges et al., 2008; Oberholzer et al., 2011). Recently, several receptor-type flagellar ACs have been shown to be specifically expressed in the procyclic stage, glycosylated, surface-exposed and catalytically active. Interestingly, these cyclases were differentially distributed: either along the entire flagellum or localized to just the tip of the flagellum (Saada et al., 2014). This indicates a microdomain flagellar cyclic AMP signaling in *T. brucei*, and that ACs have specific subdomains. These possibilities were further strengthened by the findings that one of these insect stage specific ACs (adenylate cyclase 6) is responsible for social motility and that functional mutation or RNAi knock-down results in a hypersocial phenotype (Lopez et al., 2015), again demonstrating the involvement of cAMP signaling in response to extracellular stimuli. All these observations together, coupled with the fact that cAMP levels are significantly increased during the differentiation of *T. brucei* BSFs to procyclic forms (Vassella et al., 1997), suggests a probable role of cAMP involvement in parasite behavior and differentiation through ACs.

Similarly, *T. cruzi* ACs form dimers (D’Angelo et al., 2002) and have been implicated in the conversion of epimastigotes in the insect midgut, and later in the hindgut, into human-infectious non-proliferative metacyclic trypomastigotes (Gonzales-Perdomo et al., 1988; Fraidenraich et al., 1993; Garcia et al., 1995), a process known as metacyclogenesis that is akin to cellular differentiation of BSF *T. brucei* to procyclic forms. This event can reportedly be triggered *in vitro* by a proteolytic fragment of *α*-D-globin from the insect host’s hindgut (Fraidenraich et al., 1993), confirming a role for cAMP in mediating parasite responses to environmental changes. *In vitro* metacyclogenesis triggered by nutritional stress also caused an increase in cAMP production (and cellular content) in two phases (Hamedi et al., 2015), with a first peak rapidly following the initiation of differentiation, and a second phase of elevated cAMP associated with the adhesion of the epimastigotes that is a prerequisite for their final differentiation to metacyclic trypomastigotes (Boker and Schaub, 1984).

### Protein Kinase A

The cAMP-dependent protein kinase (PK) family or protein kinase A (PKA) is a collection of serine/threonine kinases whose activity is dependent on levels of cAMP in the cell and is one of the most studied and best known members of the PK family (Huang, 2011). The kinetoplastid genomes contain reasonably well conserved genes for catalytic and regulatory domains of PKA (Huang et al., 2006). In *T. brucei*, a 499-amino acid protein with high homology to eukaryotic regulatory subunits of PKA was identified and named TbRSU. This led to the first actual measurement of the cyclic nucleotide-dependent kinase activity in *T. brucei*. The protein has the usual two cyclic nucleotide-binding domains, which are predicted to retain all the conserved residues necessary for function, as well as a pseudo-inhibitor site, which interacts with the catalytic subunit (Shalaby et al., 2001). However, further research on the kinase activity co-immunoprecipitated with TbRSU showed that although it displayed phosphorylation activity and was also inhibited by the protein kinase inhibitor peptide (PKI), both characteristics of PKA, it was not stimulated by cAMP but was instead stimulated by cyclic guanosine monophosphate (cGMP; Shalaby et al., 2001). There is to date scant evidence of cGMP production in any of the kinetoplastid parasites, although a soluble, cytosolic guanylate cyclase activity was described in *L. donovani* (Karmakar et al., 2006), and MacLeod et al. (2008) reported that feeding cGMP (but not cAMP) to tsetse flies resulted in higher trypanosome infection rates.

The finding that cAMP signaling mediates *T. cruzi* differentiation (Flawia et al., 1997) and the fact that PKAs are the major effectors in most eukaryotic cells, led to the need to identify PKA activity in *T. cruzi*. A cAMP-stimulatable PK fraction was identified and displayed a half-maximal effect at approximately 1 nM cAMP. As expected for a cAMP-dependent kinase its activity was not affected by cGMP; moreover, its phosphate-acceptor profile (including histones and kemptide, but not casein and phosvitin) was consistent with other PKA activities (Ulloa et al., 1988). The holoenzyme appeared to consist of two regulatory and two catalytic subunits (Ochatt et al., 1993). Expression of both the *T. cruzi* PKA catalytic subunit (TcPKAc) and the *T. cruzi* PKA regulatory subunit (TcPKAr) is similarly regulated and leads to coordinated expression in the life cycle stages, indicating that the two subunits are associated *in vivo*, as also shown by immunoprecipitation of the holoenzyme (Huang et al., 2002; Huang, 2011). TcPKAc activity was inhibited by the PKA-specific inhibitor PKI and both TcPKAc and TcPKAr localized to the plasma membrane and the flagellar region (Huang et al., 2002, 2006; Bao et al., 2010). TcPKAr was found to interact with several P-type ATPases, which suggests that these P-type ATPases may play a role in anchoring PKA to the plasma membrane and could play a role in compartmentalization of the kinase (Bao et al., 2009), as reported for some mammalian P-type ATPases (Xie and Cai, 2003).

The functional importance of the TcPKAc in *T. cruzi* was examined by introducing a gene encoding a PKI peptide containing a specific PKA pseudo-substrate, Arg-Arg-Asn-Ala, into epimastigotes. Expression of this PKI has a lethal effect on the parasite. Similarly, a pharmacological inhibitor, H89, killed epimastigotes at a concentration of 10 μM proving that PKA enzymatic activity is essential for the survival of the parasites (Bao et al., 2008). A yeast two hybrid screen for the substrates of PKA identified 38 candidate proteins that interact with TcPKAc, including eight genes with potential regulatory functions with respect to environmental adaptation and differentiation. These included a type III PI3 kinase (Vps34), a putative PI3 kinase, a MAPK, a cAMP-specific phosphodiesterase (PDEC2), a hexokinase, a putative ATPase, a DNA excision repair protein and an aquaporin. PKA phosphorylated the recombinant proteins of these genes (Bao et al., 2008). Additional findings also suggest TcPKAc may play a role in invading cells by mediating protein trafficking that enables parasite adhesion to cells, enabling the invasion thereof, as *trans*-sialidases were found to be substrates of TcPKA (Bao et al., 2010). As discussed by Huang (2011), there seems to be co-incidence of cAMP production, PKA activity and *trans*-sialidase expression enabling the differentiation from late stage epimastigotes to invasive trypomastigotes—all consistent with a role for cAMP signaling in differentiation and invasion by *T. cruzi*.

A *Leishmania* catalytic subunit of PKA (LdPKA) was first isolated and characterized from *L. donovani* promastigotes by column chromatography and found to be similarly inhibited by PKI as in *T. brucei* and *T. cruzi*, indicating that the kinetoplastid enzymes are likely to be structurally related, as well as topologically similar to mammalian PKA. Indeed, LdPKAc was able to make a functional holoenzyme when combined with the regulatory subunit of a mammalian cAMP-dependent kinase (Banerjee and Sarkar, 1992). In *Leishmania major*, a gene encoding a protein with high homology to other PKA catalytic subunits (LmPKA-C1) was cloned. Analysis of the sequence and structural modeling showed the protein to have all the conserved domains of eukaryotic PKAs involved in ATP and substrate binding. However, some structural and functional differences were observed with other PKA-C subunits, such as a unique 8-residue C-terminal extension (Siman-Tov et al., 1996, 2002). Expression of LmPKA-C1 was developmentally regulated with expression barely detectable in intracellular amastigotes, in contrast to a high expression level in insect-stage promastigotes (Siman-Tov et al., 1996; Duncan et al., 2001).

The role of cyclic nucleotide-regulated PK activities in promastigote proliferation and infectivity was confirmed in *Leishmania amazonensis*, with PKA activity particularly high in metacyclic promastigotes, which are primed for macrophage invasion (Genestra et al., 2004). PKA inhibitors PKI and H89 affected both replication and macrophage infection. Smaller effects were observed with the PDE inhibitors dipyridamole, rolipram and isobutyl-methyl-xanthine (IBMX) but to date we are not aware of confirmation that these effects were mediated by one of the leishmanial PDEs, or which one. These effects were temporary and did not affect intra-macrophage growth (Malki-Feldman and Jaffe, 2009).

### Phosphodiesterases

It is long been self-evident that increased knowledge of cyclic nucleotide signaling pathways can lead to the development of therapeutic agents against human diseases (Maurice et al., 2014). General pharmacological principles particularly support the potential of PDEs as therapeutic targets, as regulating the degradation of a second messenger or ligand offers a more effective intervention in cellular levels than through regulation of the rate of synthesis. Moreover, endogenous levels of the substrates (cAMP and cGMP) are not very high within the cells (between submicromolar and at most 10 μM) and competitive inhibitors can therefore be much more effective than against, for instance, PKs, where inhibitors must compete against millimolar levels of ATP (Bender and Beavo, 2006).

In mammals, PDEs exist as a superfamily and are classified into 11 families on the basis of their sequence identity, biochemical and pharmacological properties, regulation, and substrate specificity (Maurice et al., 2014). PDEs have their well-conserved catalytic domain located near their C-terminus and may contain various regulatory domains at the N-terminal end (Laxman and Beavo, 2007; Shakur et al., 2011) which presents extensive variations (Gancedo, 2013). PDEs may contain allosteric cyclic nucleotide binding sites in addition to their catalytic sites.

Phosphodiesterases have been grouped into three classes based on their different catalytic domains. Class I PDEs are found in all eukaryotes and they are the only forms of PDEs in higher eukaryotes (Beavo, 1995). Class I PDEs are the only enzymes that are capable of efficiently hydrolysing cyclic nucleotides. The genome of known kinetoplastids encodes four different class I PDEs (PDE-A to PDE-D) and does not contain members of the other PDE classes (Beavo, 1995) just as is the case in the human genome (Seebeck et al., 2011). At least one copy of each of the four PDE genes is present in the genome database of *T. brucei*, *T. cruzi*, and *L. major* (Vij et al., 2014). Class II PDEs are found in certain prokaryotes (e.g., *Vibrio fischeri*) or fungi (e.g., *Saccharomyces*, *Candida*) and in many lower eukaryotes (e.g., *Dictyostelium discoideum*). These PDEs also catalyze the hydrolysis of phosphodiester bonds but they do not show the same substrate selectivity as the class I enzymes (Bender and Beavo, 2006). Class III PDEs are restricted to the bacteria (Gancedo, 2013).

Phosphodiesterases are regulated at multiple levels and by a number of factors, such as at the genetic level (transcriptional control), through biochemical mechanisms (e.g., phosphorylation and dephosphorylation), binding of Ca^2+^, various protein–protein interactions, and by binding of cAMP or cGMP to allosteric sites (Bender and Beavo, 2006). The field of PDE research has greatly advanced and moved from basic identification of PDE enzymes and characterization of their kinetic and regulatory properties to more recent work on their structure and activity regulation. Major efforts, and important successes, are ongoing in the pharmacological exploitation of human PDEs (Azam and Tripuraneni, 2014; Chen et al., 2015; Fallah, 2015). In contrast, after 30 years of work in the area of cAMP signaling and its role in the cell biology and virulence of kinetoplastids, many of the fundamental questions remain unanswered. Shakur et al. (2011) argue that the implicit assumption that cAMP signaling in kinetoplastids would be organized as in mammals substantially delayed progress. Although this assumption has proven to be far from true, the catalytic domains of trypanosomatid PDEs, at least, are as highly conserved in relation to their human homologs as the 11 human PDEs are among themselves and similarly are suitable targets for drug screening and development (Seebeck et al., 2011; Shakur et al., 2011).

**FIGURE 1:**
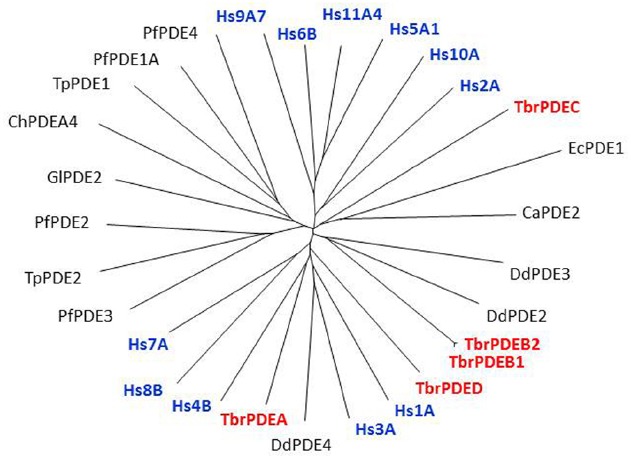
**Non-rooted tree of Class I protozoan and human PDEs.** The catalytic domains of many protozoan PDEs, including *T. brucei* (in red), are as closely related to the human PDEs (in blue) as these are among themselves. Hs, *Homo sapiens*; Pf, *Plasmodium falciparum*; Ca, *Candida albicans*; Tp, *Theileria parva*; Gl, *Giardia lamblia*; Ch, *Chilomastix hominis*; Ec, *Encephalitozoon cuniculi*; Dd, *Dictyostelium discoideum*. Figure courtesy of Professor T. Seebeck, University of Bern, Switzerland.

The kinetoplastid genomes all code for the same set of cyclic nucleotide-specific class 1-type PDEs with catalytic domains that are highly similar to those of the human PDEs (Figure 1; Beavo, 1995; Kunz et al., 2006). PDEs are hydrolases that convert cAMP or cGMP into the corresponding 5′-monophosphates (5′-AMP and 5′-GMP; Alonso et al., 2006). This makes them important players in signaling pathways as they regulate the (rate of) degradation of these cyclic nucleotides, and are thus an important factor in determining cyclic nucleotide concentrations at the cellular and subcellular levels (Bender and Beavo, 2006). Through their own cellular distribution PDEs can be instrumental in directing or containing a cyclic nucleotide signal in a particular location, thereby preventing its diffusion throughout the cell (Johner et al., 2006; Maurice et al., 2014).

Although protozoan PDEs are valid targets for the development of antiparasitic drugs, one must not ignore the potential of side-effects arising from inhibition of human PDEs. Early work, testing mammalian PDE inhibitors against kinetoplastid PDEs was encouraging in that these displayed no significant activity against the parasite enzymes (Johner et al., 2006; Laxman et al., 2006; de Koning et al., 2012), suggesting that they are pharmacologically distinct from mammalian PDEs and that structure-assisted design of selective inhibitors should be possible, just as it has been for single human PDEs. Although the fine-tuning of an inhibitor to a single therapeutic target could aid in the development of drug resistance by single point mutations in the target enzyme, this is an unfortunate reality in all target-based drug design (Seebeck et al., 2011). However, as the inhibitors are targeted to the active site of essential enzymes (protozoan PDEs), mutations that also reduce substrate binding or catalytic activity would be lethal to the parasites. *In vitro* induction of resistance to the PDE inhibitor CpdA in *T. brucei* did not result in PDE mutations (Gould et al., 2013).

In *T. brucei*, PDEA is a single-copy gene; apart from the Class I active domain it shows almost no similarity to the mammalian PDEs, placing it in a separate gene family, and appears to be expressed throughout the life cycle (Kunz et al., 2004). It has been characterized but does not appear to be essential for BSFs of *T. brucei*, as genetic deletion mutants were viable and did not display reduced proliferation rates *in vitro* (Gong et al., 2001; Kunz et al., 2004). TbrPDEB, on the other hand, exists as a small family of genes that are much more closely related to the mammalian PDEs. The TbrPDEB family was the first kinetoplastid PDE to be cloned and characterized. Based on inhibitor studies, TbrPDEB1 was believed to be an essential protein for the proliferation of the African trypanosomiasis parasite and regulation of cyclic nucleotide levels (Zoraghi and Seebeck, 2002). However, the parasite expresses two closely related PDEB alleles, TbrPDEB1 and TbrPDEB2, which can compensate for each other; knockdown by RNAi of both alleles together leads to severe cell cycle defects and cell death, both *in vitro* and *in vivo* (Oberholzer et al., 2007). Interestingly, the two isoforms have somewhat different cellular localizations. Whereas, TbrPDEB1 is located only in the flagellum, TbrPDEB2 additionally localizes to the cytoplasm (Oberholzer et al., 2007). As knockdown of TbrPDEB2 alone does not affect cellular viability it must be the loss of flagellar PDE activity that is critical.

**FIGURE 2:**
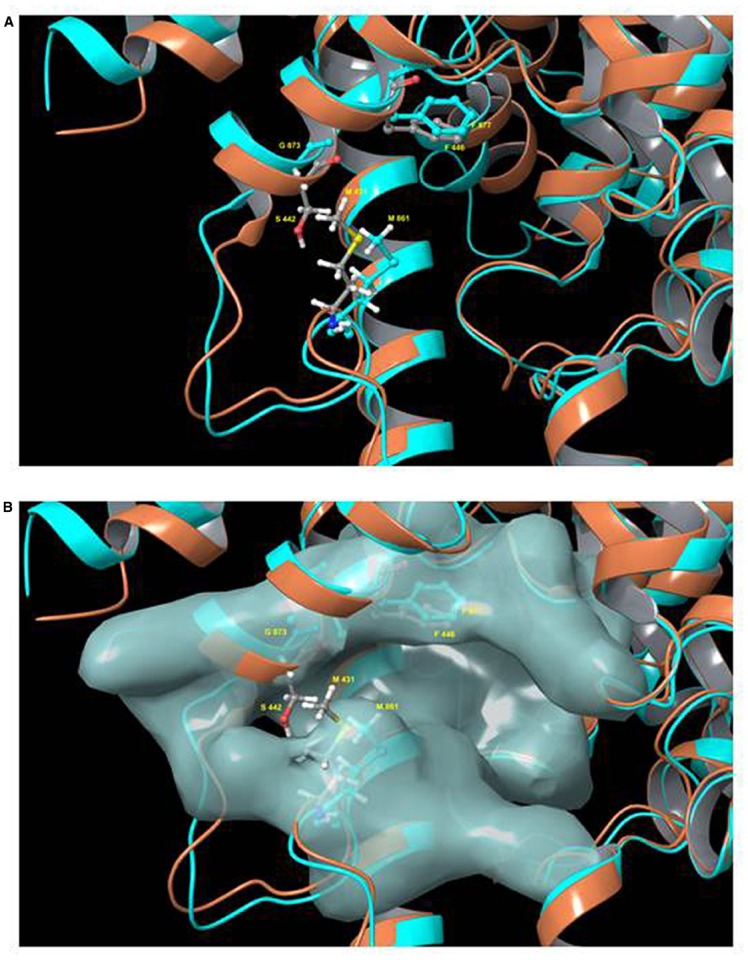
**Model of the binding pocket of TbrPDEB1 and hPDE4.** Model of the superimposed binding pockets of TbrPDEB1 (turquoise ribbons and carbon atoms) and hPDE4B (orange ribbons, gray carbon atoms). The figure depicts chain A from the published 4I15 PDEB1 structure and chain B of hPDEB structure 1XM4 (alignment RMSD 1.847 Angstrom). **(A)** Ribbon model of the cAMP binding pocket. **(B)** Same view but with the molecular surface for TbrPDEB1 residues shown. Side chains for the conserved hydrophobic clamp phenylalanine residue in TbrPDEB1 (Phe877, turquoise) and hPDE4B (Phe446, gray carbons) are shown to illustrate the orientation of the P pocket relative to this canonical binding site feature. Side chains for the pair of amino acid residues at the entrance to the P pocket in TbrPDEB1 and hPDE4B are also shown—Met861 and Gly873 in TbrPDEB1 (turquoise), and Met431 and Ser442 in hPDE4B (colored by element—carbon gray, hydrogen white, nitrogen blue, oxygen red, sulfur yellow). For TbrPDEB1 the P-pocket is clearly visible in Frame B, directly adjacent to the main ligand binding site and delineated by M861 and G873, where in hPDE4B this space is filled entirely by M431 and S442. The models were constructed by Dr. R. K. Campbell of the Marine Biology Laboratory, Woods Hole, MA, USA, using Maestro software release 2015-2 (Schrödinger, Portland, OR, USA).

More recently, a tetrahydrophthalazinone compound named CpdA, a highly potent inhibitor of both TbrPDEB isoforms, was shown to display similarly potent activity against the parasites *in vitro*. The inhibitor was discovered from a screen of more than 400,000 compounds and displayed an IC_50_ value below 10 nM against TbrPDEB1, with similar activity on TbrPDEB2, and a mid-nanomolar effect on trypanosome viability (de Koning et al., 2012). Independent pharmacological validation of the TbrPDEB isoforms was also reported by Bland et al. (2011) using the hPDE4 inhibitor piclamilast and a number of analogs. As CpdA was also known to be a potent inhibitor of human PDE4 (Van der Mey et al., 2001a,b), it is clear that the TbrPDEB family is pharmacologically closest to this human PDE. In contrast, human PDE5 inhibitors including sildenafil and tadalafil analogs displayed only weak inhibition of TbrPDEB1 (Ochiana et al., 2012; Wang et al., 2012a). Validation of the pharmacological importance of TbrPDEB1 was further confirmed when homology modeling and docking studies were used to guide fragments of Catechol Pyrazolinones into the parasite pocket (P-pocket) of TbrPDEB1 resulting in a new series of compounds with nanomolar EC_50_ values against the enzyme while also displaying promising trypanocidal activity and stimulating cellular cAMP levels (Orrling et al., 2012). The presence of the P-pocket in the otherwise highly conserved cAMP binding site was first reported for *L. major* PDEB1 (Wang et al., 2007) but present in all members of the kinetoplastid PDEB family examined to date, including TbrPDEB1 (Jansen et al., 2013), as well as TcrPDEC (Wang et al., 2012b). This is obviously very important since it allows for the development of kinetoplastid-specific PDE inhibitors with minimal or no cross reactivity with mammalian PDEs, which lack this pocket (Figure 2).

**FIGURE 3:**
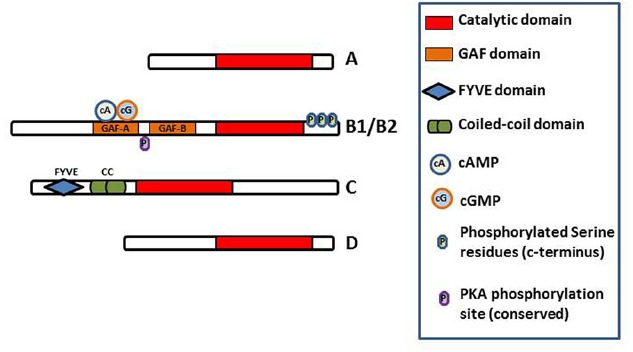
**Diagram of the domain structure of the kinetoplastid PDEs.** The conserved catalytic domain is the main functional domain of the PDEs and binds cAMP. The GAF-A domains of PDEB1 and B2 bind cAMP and cGMP and regulate the function of the catalytic domain (Laxman et al., 2005). FYVE finger has been shown to bind two Zn^2+^ ions. Coiled-coil domains are important in stabilizing protein structure and thus for protein function. Phosphorylation of the indicated serine residues of PDEB1 has been observed in the *T. brucei* phosphoproteome (Nett et al., 2009) whilst a probably functionally conserved PKA phosphorylation site is predicted in PDEB1/B2 (Shakur et al., 2011).

In *T. cruzi*, TcrPDEB1 was located in membrane fractions of the parasite and confocal microscopy showed it to be strongly associated with the flagellum (D’Angelo et al., 2004). The very high level of homology between kinetoplastid PDEB genes, and the conserved duplication into a B1 and B2 allele in tandem, appear to indicate that these genes play a crucial regulatory function in the cells. All kinetoplastid PDEB family members contain two N-terminal cAMP-binding GAF regulatory domains and a C-terminal catalytic domain (Laxman et al., 2005; Diaz-Benjumea et al., 2006; Johner et al., 2006; Shakur et al., 2011), but none of the other kinetoplastid PDE families do (Figure 3). Of these PDE families, the Phosphodiesterase D (PDEDs) have as yet barely been explored beyond the mere presence of the homologous genes in the respective genomes.

As at least some of the trypanosomatid PDEs have been shown to be essential regulatory enzymes, there is now much interest in these enzymes as drug targets and substantial efforts are ongoing, ranging from high-throughput screening, to structure-based design, compound repurposing and fragment-based inhibitor design (de Koning et al., 2012; Amata et al., 2014, 2015; Blaazer et al., 2015). As discussed extensively by Seebeck et al. (2011), this strategy has many advantages to drug development for the highly NTDs caused by kinetoplastid parasites, exactly because the target is highly conserved with closely related human homologs. First of all the interest in human PDEs by Big Pharma has resulted in large compound libraries of potential inhibitors. Furthermore, the potential side-effects and toxicity issues of inhibiting any of the human PDEs have been well investigated, as have the stability and pharmacokinetic properties of most of the inhibitor scaffolds. Most crucially, it has proven to be relatively straightforward to engage with the pharmaceutical industry on inhibitors for kinetoplastid PDEs, as they already have similar programs for other diseases. This strategy has allowed the rapid identification of potent inhibitors of TbrPDEB and other kinetoplastid PDEs but needs to rely on relatively small differences in the binding pocket, notably the P-pocket (Figure 2) of the enzyme, to achieve selectivity over human PDEs.

## The Role of cAMP Signaling in *T. brucei*

The presence of cAMP in trypanosomes, and its variation during the course of infection, were recognized early on (Strickler and Patton, 1975). However, the completion of various kinetoplastid genome projects has revealed that cAMP signaling in the kinetoplastids is starkly different from the pathways so extensively studied in higher eukaryotes. Some important differences include the fact that kinetoplastid genomes do not code for G-protein-coupled receptors, or for heterotrimeric G proteins or PK G. Furthermore, the ACs are structurally very different from their mammalian counterparts, although the basic catalytic mechanism seems to be conserved between them, and may have assumed the role of receptors. Finally, apart from apparent PKA subunits in *T. cruzi* (Huang et al., 2006) and *L. donovani* (Bhattacharya et al., 2012), no homologous genes for cAMP effectors were identified in these genomes. The regulatory subunit of *T. brucei* PKA does not appear to bind cAMP, but instead binds cGMP (Shalaby et al., 2001), although, as discussed above, there is no convincing evidence that cGMP is produced by these parasites. Thus, it is likely that PKA is not activated by cyclic nucleotides in *T. brucei*.

The need for parasite survival implicates the need for a developmental response to adapt to different environments encountered within the mammalian host and throughout the arthropod vector. This is especially so during preparation for transmission, where specialized developmental forms are often generated to promote survival when ingested or propagated by a biting insect (Baker, 2010; MacGregor et al., 2012). The result is a dynamic balance of transmissible and proliferative stages within a host, ensuring that the population can maximize its longevity within the host but also optimize its capacity for spread to new hosts (Mony et al., 2014). The morphotypes that characterize a certain genus are cell shape, dimensions and the positions of the kinetoplast-flagellar pocket relative to the nucleus (Svobodova et al., 2007). These complex morphological and biochemical changes during cell differentiation in trypanosomatids are environmentally driven through different ligands and/or stimulatory molecules present in these environments, most of which are yet to be identified (Parsons and Ruben, 2000).

The report of AC activity in *T. b. gambiense* in 1974 (Walter et al., 1974) implicated the possible role of cAMP signaling in the cell biology and virulence of kinetoplastids, triggering the study of cAMP levels in the different life cycle stages of *Trypanosoma lewisi* (Strickler and Patton, 1975), *T. b. brucei* (Mancini and Patton, 1981), and *L. donovani* (Walter et al., 1978). Trypanosomes living in the bloodstream proliferate as morphologically “slender” forms that evade host immunity by antigenic variation, generating characteristic waves of infection. As each wave of parasitaemia ascends, slender forms stop proliferating and undergo transformation to stumpy forms, the parasite’s adaptation for transmission to the tsetse fly vector (Vickerman, 1985). Earlier cAMP measurements showed increased cAMP levels in long slender BSFs during the cyclical wave of proliferation of these cells, and relatively low cellular cAMP concentrations when the abundance of stumpy forms increases (Mancini and Patton, 1981). The differentiation of longer slender forms to short stumpy forms has been shown to be density dependent (Vassella et al., 1997) with resemblance to quorum-sensing systems found in microbial communities (Waters and Bassler, 2005). The response to this density-dependent differentiation is triggered by a yet to be identified low molecular weight molecule called SIF, and it has been speculated that this triggers a cAMP response as the cAMP analog 8-(4-chlorophenylthio)-cAMP (8-pCPT-cAMP) was demonstrated to have the same differentiation-inducing effect as SIF. Additionally, trypanosomes incubated with a conditioned medium containing SIF displayed a twofold to threefold increase in the intracellular concentration of cAMP compared with cells grown in a non-conditioned medium (Vassella et al., 1997; Breidbach et al., 2002). Moreover, there had been a tentative link between cAMP signaling and differentiation of the BSFs to the insect procyclic forms, a process that is accompanied by the shedding of its VSG surface coat (Barry and McCulloch, 2001). However, monitoring of AC activity and VSG shedding after triggering differentiation to procyclic forms showed that AC stimulation was not responsible for the release of VSG (Rolin et al., 1993), and cAMP was not required for differentiation to occur (Strickler and Patton, 1975; Mancini and Patton, 1981). High concentrations of extracellular cAMP, 5′-AMP or adenosine did not significantly affect the proliferation of *T. brucei*, suggesting that the antiproliferative effect caused by the nucleotide analogs was mediated by an intracellular “receptor.” And although 8-pCPT-cAMP did induce differentiation into stumpy-like non-proliferative forms, a hydrolysis-resistant analog did not, whereas the hydrolysis products of 8-pCPT-cAMP (i.e., the equivalent AMP and adenosine analogs) had a more potent effect than 8-pCPT-cAMP itself (Laxman et al., 2006). The clear conclusions of this study were that (1) cAMP is not the primary effector of the differentiation signal and (2) the hydrolysis products of 8-pCPT-cAMP trigger a differentiation-like transformation in *T. brucei* long-slender BSFs.

This insight was used to good effect when a genome-wide RNAi target sequencing (RITseq) approach was used to identify signaling components driving stumpy formation by exposing and selecting proliferative monomorphic cell lines unresponsive to 8-pCPT-cAMP or 8-pCPT-2-*O*-methyl-5-AMP-driven stumpy formation. This led to the identification of a cohort of genes implicated in each step of the signaling pathway, from genes involved in purine metabolism and signal transduction (kinases, phosphatases) to gene expression regulators (Mony et al., 2014). Identified genes at each step of the signaling pathway were independently validated in cells naturally capable of stumpy formation, confirming their role in density sensing *in vivo*. The putative RNA-binding protein, RBP7, was required for normal quorum sensing and promoted cell-cycle arrest and transmission competence when overexpressed. Thus, quorum sensing signaling in trypanosomes shares similarities to fundamental quiescence pathways in eukaryotic cells, its components providing targets for quorum-sensing interference-based therapeutics (Mony et al., 2014).

While a direct role for cAMP in the initiation of trypanosome differentiation events has thus become more doubtful, the role and importance of cAMP in flagellar motility and signaling is increasingly being dissected, with interesting findings. For example it is commonly believed that the flagellum, as an important host-parasite interface, has essential sensory functions (Tetley and Vickerman, 1985; Rotureau et al., 2009). For example in *Chlamydomonas reinhardtii*, the triggering of zygote formation is initiated by cAMP signaling in response to flagellum adhesion in gametes (Pan and Snell, 2000). Recently, it has been shown that cAMP regulates social motility in procyclic *T. brucei*, with social motility absent when TbrPDEB1 was inhibited by CpdA or knocked down with RNAi. The reduction in PDEB activity appeared to disrupt the generation of an extracellular signal necessary for the behavior, as the social motility was completely restored in mixed TbrPDEB1 knockdown and wild-type cells (Oberholzer et al., 2015). This is similar to social motility observation in *D. discoideum* where cAMP signaling is critical for surface motility (Firtel and Meili, 2000). It is believed that the social motility exhibited by the procyclic forms is essential for their migration from the tsetse midgut to the insect’s salivary gland, which allows it to complete it life cycle.

In BSFs of *T. brucei*, the most unambiguous role of cAMP is in cytokinesis, as either the knockdown of ACs (Salmon et al., 2012a), knockdown of TbrPDEB1 and B2 (Oberholzer et al., 2007) or the pharmacological inhibition of these PDEs (de Koning et al., 2012) all lead to severe defects in the cytokinesis phase of cell division, resulting in misshaped cells with multiple nuclei and kinetoplasts, that are ultimately non-viable.

## Novel Downstream Effectors of cAMP in Trypanosomes

Although cAMP has thus been implicated in important cellular functions and behavior of trypanosomes, the effectors that mediate this regulatory activity have been elusive. As noted above, the only potential effector protein identified, PKA, was not responsive to cAMP (Shalaby et al., 2001), and it became clear that, instead of searching for mammalian homologs, an unbiased approach to identify novel effector proteins was required. Accordingly, Gould et al. (2013) generated two CpdA-resistant lines. The first method involved exposing wild-type *T. b. brucei* trypanosomes briefly to the mutagen methyl methanesulfonate (MMS; Sigma), followed by culture in increasing but sub-lethal concentrations of CpdA. The second method employed the use of a genome-wide *T. b. brucei* RNAi library (Alsford et al., 2011, 2012; Baker et al., 2011) to select for resistance under CpdA pressure. This screen revealed four distinct genes that were knocked down, which were designated cAMP Response Proteins (CARP1–4; Gould et al., 2013). Targeted RNAi knockdown of these CARPs confirmed a significant increase in resistance to CpdA and to elevated cellular cAMP levels, confirming that they are genuine downstream effectors of cAMP signaling. One of the genes knocked down in the CpdA-resistant cultures was Tb427tmp.01.7890 (*CARP1*; Tb927.11.16210 in *T. b. brucei* reference strain TREU 927), encoding a 705-amino-acid protein containing two apparently intact and one partial cyclic AMP binding-like domain, that is conserved in synteny in each of the kinetoplastid genomes sequenced. Recently, the homolog of CARP1 in *T. cruzi* TcCLB.508523.80 has been reported to bind cyclic nucleotides, using cAMP and cGMP displacement assays (Jäger et al., 2014), further validating the role of CARP1 as a downstream cAMP signaling effector. CARP2–4 are proteins of as yet unknown functions but some of them have a probable flagellar localization, consistent with a role in mediating or regulating a cAMP signal (Gould et al., 2013).

## Summary and Outlook

**FIGURE 4:**
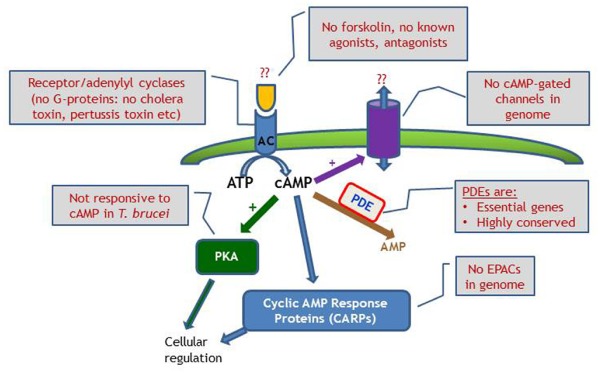
**Schematic diagram of cyclic nucleotide signaling in ***T. brucei***, emphasizing the lack of investigative tools compared with the classical mammalian model, where manipulation of receptors, G-proteins and cyclases are all possible.** EPAC, exchange protein directly activated by cAMP.

Differences in cAMP signaling between the mammalian system and trypanosome are well documented, such as the many and varied AC; no GPCRs or G-proteins; inactive PKA in *T. brucei*; and as yet to be identified AC triggers (Figure 4). However, from these differences opportunities may arise, as the downstream effects as well as the cAMP modulating receptor ligands appear to be unique to kinetoplastid parasites, and may offer promising targets for therapeutic intervention. The cAMP PDEs are already the focus of considerable drug development programs at the interface of academic research and pharmaceutical industry^1^^,^^2^.

It is believed that as further studies of the downstream effectors progress, many more similarities and/or differences with mammalian regulatory pathways will come to the fore, which will provide much needed insights into these important biological processes in eukaryotic pathogens. This is even more so as studies of cAMP signaling and its associated effect on flagellar function and social motility is increasingly revealing particularly important cellular activities of the trypanosome. The importance of the flagellum to the trypanosome and how it interacts with its environment cannot be overstated. Thus, trypanosomal cAMP signaling, and the role of the flagellum therein, offer a ready and important biological system for much needed, innovative strategies for antiprotozoal drug development.

### Conflict of Interest Statement

The authors declare that the research was conducted in the absence of any commercial or financial relationships that could be construed as a potential conflict of interest.

## Footnotes

1 http://www.openlabfoundation.org/research/projects/details12.html

2 http://www.tipharma.com/pharmaceutical-research-projects/neglected-diseases/phosphodiesterase-inhibitors-for-neglected-parasitic-diseases-pde4npd.html
